# An Evaluation of the Psychometric Properties of the Temporal Satisfaction With Life Scale

**DOI:** 10.3389/fpsyg.2022.795478

**Published:** 2022-04-14

**Authors:** Joline Guitard, Aaron Jarden, Rebecca Jarden, Denis Lajoie

**Affiliations:** ^1^School of Psychology, Université de Moncton, Moncton, NB, Canada; ^2^Center for Wellbeing Science, Melbourne Graduate School of Education, University of Melbourne, Melbourne, VIC, Australia; ^3^Department of Nursing, Melbourne School of Health Science, University of Melbourne, Melbourne, VIC, Australia

**Keywords:** life satisfaction, assessment, psychometric, wellbeing, positive assessment

## Abstract

The Temporal Satisfaction with Life Scale measures judgements of life satisfaction using 15 items, according to three temporal dimensions: past, present, and future. However, only seven studies have looked at the psychometric properties of the Temporal Satisfaction with Life Scale, and this has been individually across vastly different countries and cultures (Canada, China, Germany, Spain, Switzerland, Turkey, and United-States), and with different populations, such as undergraduate students, adults, and older adults. In addition, these studies have highlighted issues regarding the replicability of the validity of the scale structure and optimal number of items. In this study we use a large international and multicultural sample (*n* = 6,912) from the International Wellbeing Study and investigate the scale structure of the Temporal Satisfaction with Life Scale, resulting in the recommendation that a shortened 12-item version provides a better model fit compared to the original 15-item version. More in-depth correlates with aspects of wellbeing and illbeing, in relation to past, present, and future life satisfaction, are also presented than have been previously, which found positive correlations between the temporal dimensions of the Temporal Satisfaction with Life Scale and wellbeing, as well as negative correlations with illbeing measures.

## Introduction

Life satisfaction is amongst the most used concepts to assess subjective wellbeing (SWB). In fact, some authors use life satisfaction and subjective wellbeing as interchangeable synonyms (i.e., Bertoni and Corazzini, 2018). Life satisfaction can be defined as a cognitive evaluation of one's overall satisfaction with their current life, relative to one's own criteria regarding what a satisfactory life means (Diener et al., 1985). The most widely used measure of life satisfaction is the Satisfaction with Life Scale (SWLS; Diener et al., 1985), which assesses individuals' life satisfaction with five items answered on 7-point Likert scales. From this unidimensional scale, Pavot et al. (1998) developed a multidimensional measure to assess life satisfaction over time: The Temporal Satisfaction with Life Scale (TSWLS; Pavot et al., 1998). The TSWLS measures judgements of life satisfaction using 15 items, according to three temporal dimensions: past, present, and future. To create this new measure, the authors kept the original five items of the SWLS to assess present life satisfaction. Then, to create the past and future dimensions, the authors simply added words like “in the past” or “for the future” to the original five questions. For example, the item “I am satisfied with my life” which is used to measure present life satisfaction was transformed to “I am satisfied with my life in the past” to measure past life satisfaction and to “I will be satisfied with my life in the future” to measure future life satisfaction (Pavot et al., 1998). Therefore, the temporal aspects of the TSWLS aims at assessing one's current subjective perception of their life in the past, present, and expectations for the future. Pavot (2014) argues that this conceptualization is important as our present wellbeing is likely to be impacted by what we subjectively recall from the past and expect for our future (regardless of if these interpretations and expectations are accurate or not).

Since its creation, the TSWLS has rarely been used in research compared to the SWLS. For example, a search in October of 2021 for the SWLS on PsycInfo yielded 10,886 results, while a search for the TSWLS yielded only 61 results (i.e., for every one time the TSWLS is used in research, the SWLS is used 178 times). However, when assessing life satisfaction, researchers may benefit from the temporal dimensions of the TSWLS as it provides more specific information regarding individual differences in levels and experiences of wellbeing (Pavot et al., 1998). For example, let's imagine two individuals who answer the SWLS, and both get an average score of 20. During data analysis, these two individuals would be considered to have the same level of life satisfaction, even if one of them expects their life satisfaction to be better in the future and the other believes their life satisfaction to be worst in the future. Such differences in one's vision of future life satisfaction could have substantial impacts on research (i.e., misinterpretation of results), especially if life satisfaction is used as an independent variable to predict concepts that imply a future-oriented perspective (e.g., optimism, hope; Pavot et al., 1998). The explanation illustrated by the example above also applies to the past dimension of the TSWLS and concepts that imply a past-oriented perspective (i.e., depression, rumination). Furthermore, differences in how one recalls their past or envisions their future can have impact on motivations and coping strategies (Pavot et al., 1998).

In addition to having been used less often than the SWLS, the TSWLS's psychometric properties have rarely been investigated. Specifically, only eight studies, including the original study by Pavot et al. (1998), have investigated the psychometric properties of this scale, and this has been across vastly different cultures and languages in the different countries of Turkey (Akyurek et al., 2019), Canada (McIntosh's, 2001), China (Ye, 2007), Germany (Trautwein, 2004), Spain (Tomás et al., 2016; Carrillo et al., 2021), Switzerland (Proyer et al., 2011), United-States (Pavot et al., 1998), and in different populations, such as undergraduate students (e.g., McIntosh's, 2001), adults (e.g., Akyurek et al., 2019), and older adults (e.g., Carrillo et al., 2021). Moreover, of those studies, only Pavot et al. (1998) and McIntosh's (2001) were conducted using the original English version; all other studies used various non-English adaptations of the scale (i.e., Chinese, German, Spanish, Swiss, Turkish). It is not explicitly known why there has not been more studies using or studying the TSWLS. However, one possible reason is that the temporal aspect of the scale often requires multiple time-points of assessment and therefore, requires more time and resources than a single time-point assessment done by the SWLS. Nevertheless, as depicted by the example above, neglecting to consider the cognitive component that underlies how individuals see their past and future life satisfaction, even in a single time-point assessment, could lead to wrongful conclusions if other study variables have an implied temporal component.

In terms of the TSWLS structure, all studies (except for Tomás et al., 2016-Spanish version) found support for a three-factor structure; factors being past, present, and future life satisfaction. Tomás et al. (2016) instead reported finding a bifactor model comprising one general dimension of satisfaction with life and three specific factors for past, present, and future life satisfaction. A more recent study of Spanish speaking individuals was conducted by Carrillo et al. (2021) who performed their own translation of the original scale into Spanish. Their results supported a three-factor structure which includes all 15 original items. The Turkish adaptation of the scale was found to have a better fit with the first item of each five-item subscale removed, resulting in a 12-item measure (Akyurek et al., 2019), whereas the Chinese adaptation was found to have a better fit with the first and last items of each 5-item subscale removed, with then each subscale resulting in 3-items and in a 9-item measure (Ye, 2007). Thus, it appears there are unresolved issues regarding the replicability of the validity of the scale structure and optimal number of items. Regarding reliability, internal consistency of the scale has been reported as good (Trautwein, 2004) and alpha coefficients have ranged from 0.87 to 0.93 (Pavot et al., 1998; Tomás et al., 2016; Akyurek et al., 2019; Carrillo et al., 2021). For the subscales, good internal consistency has also been reported with alpha coefficients ranging from 0.76 to 0.93 (Pavot et al., 1998; McIntosh's, 2001; Proyer et al., 2011; Tomás et al., 2016; Akyurek et al., 2019; Carrillo et al., 2021). Reports of test-retest reliability have also been respectable (Trautwein, 2004) with correlations between times of measurements of 0.81 (Akyurek et al., 2019) and 0.83 (Pavot et al., 1998).

## Aims and Hypotheses

The current study aims to assess the psychometric properties of the Temporal Satisfaction with Life Scale (TSWLS; Pavot et al., 1998), specifically investigating the scale structure and number of optimal items by testing for measurement invariance between subsets of our sample. More precisely, we will test for measurement invariance between English speakers of different countries as well as between six different translations of the scale. This is in relation to (a) the TSWLS's little use since its creation and little investigation (eight studies) confirming its psychometric properties, (b) the issue of replicability of the validity of the scale structure and optimal item count, and (c) extending the current research base by reporting for the first time on a large and diverse multicultural (rather than single culture) sample.

Regarding the general scale structure and optimal number of items, we are going to test which configuration (the original 15-item three factor structure or a 12-item version) receives support across different cultures given that the 12-item version has been deemed more suitable in several studies. Regarding measurement invariance, we expect to find strict invariance of the English version and at least configural invariance between the different translations. As the TSWLS is a measure of life satisfaction, we expect all three subscales to positively correlate with aspects of wellbeing (strengths use and knowledge, subjective happiness, gratitude, hope, and the presence of meaning in life) and to negatively correlate with aspects of illbeing (search for meaning in life, rumination, depression).

## Method

### Participants

The current study used data from the International Wellbeing Study (IWS; www.wellbeingstudy.com). Our sample in the current study consists of 6,912 individuals who completed the assessment battery at the first assessment timepoint of this five timepoint longitudinal study, with participants who did not complete the whole battery at this timepoint excluded and further timepoint data not included in this analysis. Individuals came from various countries and cultures such that 3,982 were English-speaking participants who completed the English version of the survey, and 2,930 were non-English speaking participants who completed translated versions of the survey in their respective languages. English speaking participants came from 89 different countries and were grouped by their world region for the purpose of analyses. Most of the English-speaking participants were from the regions of Oceania, North America, and Europe as depicted in Table 1. The distribution and descriptive statistics of non-English-speaking participants according to their language of assessment is available in Table 2. Data was collected between March 2009 and March 2013. All participants were over the age of 16 (81.5% female: mean age 37.4 years old, *SD* 14.3).

**Table 1:** English-speaking participants' world region.

**World region**	**Relative frequency (%)**	**Count**
Oceania	48.4	1,928
North America	24.0	956
Europe	19.6	779
Asia	5.4	216
Africa	1.3	50
Middle East	0.8	31
Latin America	0.6	22
Total	100%	3,982

*Latin America includes countries from Central and South America as well as the Caribbeans*.

**Table 2:** Distribution and descriptive statistics of non-English-speaking participants according to their language of assessment.

**Language**	**Count**	**Mean age (*SD*)**	**% Females**	**Most represented countries per language**
Hungarian	1,136	31.5 (11.38)	84.2	1,068 (94.01%) living in Hungary
Spanish	693	35.5 (13.43)	76.8	309 (44.59%) living in Mexico; 200 (28.86%) living in Columbia
Finnish	335	20.5 (14.18)	51.6	314 (93.73%) living in Finland
Slovene	288	23.3 (9.76)	83.0	281 (97.57%) living in Slovenia
Czech	250	27.6 (10.97)	81.6	241 (96.4%) living in Czech Republic
Chinese	228	21.8 (6.73)	58.8	205 (89.91%) living in China

*n = 2,930*.

### Materials

The IWS survey battery consisted of 19 questionnaires (217 items in total) and was completed in 29 min on average. The current study uses nine of the 19 questionnaires from the IWS; each of these nine are described below, and the other 10 we did not view as direct illbeing or wellbeing correlates (e.g., Negative Life Event Scale), and also perceived they were not needed. A full list of the IWS survey battery and copy of the survey questions in each language is available on the IWS website. Regarding translations, the English version of the TSWLS was that provided by Pavot et al. (1998). For the International Wellbeing Study this scale was back-translated into 15 languages, including the six used in this study (Chinese, Czech, Finnish, Hungarian, Slovene, and Spanish). In each case the scale was first translated from English into the relevant language by a native speaker of the language, who also had psychology and scale development knowledge. The translation was then independently translated back into English by a second translator, and then the two translators discussed and resolved any inconsistencies in translation. The detailed reliability statistics are available in supplemental material for each translation of the scale, as well as for each English-speaking subsamples used in the current study.

The Temporal Satisfaction with Life Scale (TSWLS; Pavot et al., 1998) measures past, present and future life satisfaction according to 15 items (five per temporal dimension). Items are answered on 7-point Likert scale ranging from 1—*strongly disagree*, to 7—*strongly agree*. Internal consistency was deemed as mostly good with coefficients alpha and omega ranging from 0.80 to 0.87 for past life satisfaction, 0.88 to 0.91 for present life satisfaction, 0.72 to 0.87 for future life satisfaction and 0.87 to 0.94 for the total scale.

The Strengths Use and Current Knowledge scale (Govindji and Linley, 2007) consists of 10 items (five per subscale) measuring the use (e.g., “I always try to use my strengths”) and knowledge (e.g., “I know my strengths well”) of one's psychological strengths. The scale can also be used to obtain a global score of strengths use and knowledge. Items are answered on a 7-point Likert scale ranging from 1—*strongly disagree* to 7—*strongly agree*. In the current study, internal consistency coefficients ranged from 0.84 to 0.90 for the use subscale, 0.70–0.85 for the knowledge subscale and 0.86–0.92 for the total scale, showing support for acceptable to good internal consistency.

The Subjective Happiness Scale (SHS; Lyubomirsky and Lepper, 1999) is a 4-item measure of global subjective happiness designed to assess how happy individuals consider themselves to be. Items are answered on a 7-point Likert scale, with scale anchors differing across the four items. An example item is: “Compared to most of my peers, I consider myself:” with answers ranging from 1—*less happy* to 7—*more happy*. Internal consistency of this scale in the present study was adequate with coefficients alpha and omega ranging from 0.74 to 0.86, with the exception of the Finnish translation which showed poor internal consistency (α = 0.49, ω = 0.66, 95% CI [0.60, 0.72], SE = 0.03).

The Happiness Measure (HM: Fordyce, 1988), also known as the Fordyce Emotion Questionnaire, is a measure of emotional wellbeing that provides an indication of a person's perceived happiness and measures the affective component of SWB. The HM consists of two questions on happiness; the first one assessing how happy the individual usually feels (intensity), while the second is an estimate of the percentages of time respondents feel happy, unhappy, and neutral (frequency). For the purpose of the current study, only the first item was used, which asks respondents: “In general, how happy or unhappy do you usually feel?.” Respondents choose one of 11 descriptive answers ranging from (0) “extremely unhappy (utterly depressed, completely down),” to (5) “neutral (not particularly happy or unhappy),” to (10) “extremely happy (feeling ecstatic, joyous, fantastic).” With the item used in the current study, the HM measures individuals' perceptions of their intensity of happiness in general. This is in contrast to the SHS which measures more than intensity in capturing a more global and cognitive aspect of happiness (e.g., one's happiness compared to others).

The Gratitude Questionnaire-6 (GQ-6; McCullough et al., 2002) is a unidimensional 6-item measure of the disposition toward gratitude. Items are answered on a Likert scale ranging from 1—*strongly disagree* to 7—*strongly agree*. An example item is: “I am grateful to a wide variety of people.” In the current study, the GQ-6 had adequate internal consistency with coefficients alpha and omega ranging between 0.71 and 0.84.

The Adult Hope Scale (AHS; Snyder et al., 1991) measures hope according to two dimensions: agency and pathways. The agency dimension assesses successful goal-directed determination (e.g., “I energetically pursue my goals”), while the pathways dimension assesses the ability to find ways of surmounting obstacles (e.g., “There are lots of ways around any problem”). The scale consists of 12 items: four agency items, four pathway items, and four fillers not related to hope (e.g., “I feel tired most of the time”). Items are answered on an 8-point Likert scale ranging from 1—*definitely false* to 8—*definitely true*. The AHS provides scores for both dimensions (agency and pathways), as well as a global hope score based on the eight hope related items. Internal consistency for the scale in the current study was mostly acceptable with alpha and omega coefficients ranging from 0.72 to 0.84 for the agency subscale, 0.68 to 0.83 for the pathway subscale and 0.78 to 0.88 for the total scale.

The Meaning in Life Questionnaire (MLQ; Steger et al., 2006) measures the presence and the search for meaning in life with 10 items (five per subscale). The presence subscale assesses “‘individuals' feelings of living a” meaningful life (e.g., “My life has a clear sense of purpose”), whereas the search subscale assesses “‘individuals' motivations to” find or better understand the meaning in their lives (e.g., “I am looking for something that makes my life feel meaningful). Items are answered on a 7-point Likert scale ranging from 1—*absolutely untrue* to 7— *absolutely true*. In the current study, both the presence and search subscales had good internal consistency with alpha and omega coefficients ranging from 0.79 to 0.93 and 0.83 to 0.91, respectively.

The Rumination Scale used in the current study was a 6-item adaptation from the 22-item Ruminative Response Style subscale of the Response Styles Questionnaire (Treynor et al., 2003), specially created for the IWS by Professor Paul Jose. The scale assesses responses to depressive symptoms focusing on their meanings, causes and consequences (Nolen-Hoeksema, 1991). The scale consists of six items prompted with: “In the past 3 months would you say you…”. Two items came from the Brooding-related factor (or moody and self-critical pondering, e.g., “thought: ‘Why can't I handle things better?”' and four items came from the Depression-related factor (directly tapping into depression symptoms, e.g., “thought: ‘Why can't I get going?”'; Treynor et al., 2003). When put together with the prompt, an example of an item would be: “In the past 3 months, would you say you thought: ‘Why can't I handle things better?.”' Items were answered on a 7-point Likert scale ranging from 1—*strongly disagree* to 7—*strongly agree*. Internal consistency of this scale in the current study was good with coefficients ranging from 0.82 to 0.89 across subsamples.

The Centre for Epidemiological Studies Depression Scale (CES-D; Radloff, 1977) was used to assess the presence of depressive symptoms in the last week, while focusing on the affective component. The unidimensional scale consists of 20 items (e.g., “I felt tearful”) that are answered on a 4-point scale ranging from 0—*rarely or none of the time (*<*1 day)* to 3—*most or all of the time (5–7 days)*. In the current study, the CES-D had excellent internal consistency with alpha and omega coefficients ranging from 0.90 to 0.93.

### Procedure

Ethical approval was granted by the *Open Polytechnic of New Zealand* Ethics Committee in 2009. Participants started the study at different times between March 2009 and March 2012. Self-reported questionnaires were completed at ~3-month intervals (during an open week period) for a total of five assessments over a year. Incentives for participation included a summary report of their scores on the survey, the chance to win one of 15 Amazon.com vouchers (valued at $100 NZD), and the opportunity to take part in one of three online wellbeing classes for free after the first three assessments.

### Statistical Analyses

Analyses were conducted with Lavaan 0.6–9 package in R version 4.1.1 (R Core Team, 2022). We adopted the MLR estimator in our analyses. Indeed, all the items in the TSWLS are statistically non-normal in our sample (either in terms of skew, kurtosis, or both). This may not be cause for concern, as even small deviations in skew and kurtosis can appear to be statistically significant with a large sample, such as ours (Tabachnick and Fidell, 2014, p. 114). Nevertheless, adopting a robust estimator avoids potential issues with non-normality. To take full advantage of our international and multilingual sample, we adopted a multistep analytic strategy. The first step was to optimize model fit in a regional subset of our English sample. We decided to use the English version as the baseline model as it is the original language of the scale (Pavot et al., 1998) and the largest segment of our sample. The second step was to cross-validate the optimized model in the rest of the English sample (i.e., other regions) and to establish measurement invariance within that sample. The third step was to verify measurement invariance in the translations. We verify Configural (factor structure held constant), Metric (factor loadings held constant), Scalar (intercepts held constant) and Strict (error held constant) invariance. In invariance testing, models are nested such that the constraint of previous steps are included in subsequent steps (i.e., Strict invariance implies equivalent factor structure, factor loadings, intercepts, and error for examined groups). We note that the literature on statistical criteria to determine measurement invariance is not yet fully set (see Putnick and Bornstein, 2016 for a review). The usual cut-off is Cheung and Rensvold (2002) CFI criterion for assessing measurement invariance. That is to say that so long as the CFI for the nested model is not worse by more than 0.01, invariance can be claimed. However, other authors have suggested that certain parameters surrounding the tests of invariance have an important influence on the criteria to be used. For example, Rutkowski and Svetina (2014) argue that when multiple groups are compared, more liberal criteria should be used. For instance, in their analyses containing a range of 10–20 groups, they argue that a .02 reduction for CFI and a .03 augmentation for RMSEA would be evidence of invariance. Overall, then, contextualized interpretations of measurement invariance statistical criteria are preferred rather than specific cut-off points.

## Results

### Scale Structure and Measurement Invariance

Because we wanted to test measurement invariance, we split our English participants by their continent of origin. We retained regions for which at least 200 participants could be identified in our analyses (Oceania, North America, Europe, and Asia). We decided to test and optimize fit in the Oceania sample, the largest group in the English sample, with the knowledge that our results with this group would be cross-validated using the other regional groups. Because we had strong theoretical expectations regarding the overall shape of the model (multiple previous studies having established a three-factor structure), we opted for a Confirmatory Factor Analysis (CFA) framework.

The first model we tested (Model 1) was therefore a three-factor model with all 15 items. As shown in Table 3, fit was mediocre. An examination of the factor loadings for this model showed that item 11 was problematic as it did not meet the 0.6 criterion for factor loadings suggested by Awang (2012), nor did it meet the criteria of 0.4 for the R-squared values suggested by the same author. This item was therefore removed. Model 2 results represent the same model as Model 1, minus item 11. Fit remained mediocre, but was slightly improved. At this point, we made the substantive decision to test a model without items 1 and 6. These items reflect the same concept as item 11, except for the past and present timeframes, respectively. This action was motivated by the fact that we judged it important for the very concept of this questionnaire to have comparable and commensurate scores of life satisfaction across timeframes. Moreover, we determined that it would be for the best if a shorter measure contributed to better fit, as this would favor shorter response time for participants. Model 3 presents the results of a three-factor model with items 11, 1, and 6 removed. Fit was improved but did not yet meet all criteria for excellent fit. We therefore continued to examine potential theoretically based modifications that we could make to the model and concluded that it would be necessary to correlate residuals between items referring to similar concepts across timeframes. The practice of correlating residuals is controversial, but some authors have argued that excluding residual correlations that reflect real shared method variance leads to biased factor estimations (e.g., Cole et al., 2007). In the case of the TSWLS, shared method variance is included by design, with similar items used for all three temporal conditions. Model 4 therefore presents the results of a model with items 11, 1, and 6 removed, and with correlated residuals between similar items (e.g., items 2, 7, and 12; and items 3, 8, and 13). As shown in Table 3, fit was quite good for this model. We note that we did test a bifactor model (Model 4 with an added general factor with loadings on all items, and orthogonality between latent variables), but that it caused convergence issues in invariance models (and in one of the translation subsamples). We therefore excluded this analysis from our presentation.

**Table 3:** Model fit using the Oceania sample across 4 models tested.

**Model**	**Robust X2**	**Df**	**Robust CFI**	**Robust TLI**	**Robust RMSEA**	**SRMR**	**BIC**	**AIC**
Model 1	1274.363	87	0.916	0.899	0.084	0.06	94246.358	93979.274
Model 2	985.242	74	0.931	0.916	0.08	0.039	87256.214	87005.823
Model 3	672.553	51	0.942	0.925	0.08	0.035	74038.989	73821.984
Model 4	289.078	39	0.977	0.961	0.058	0.031	73600.289	73316.513

*Excellent fit can be defined by Irwing and Hughes' (2018) criteria: Root Mean Square Error of Approximation (RMSEA) ≤ 0.06, Standardized Root Mean Square Residuals (SRMR) ≤ 0.08, Tucker-Lewis Index (TLI) ≥ 0.95, Comparative Fit Index (CFI) ≥ 0.95. Abad et al. (2011) also suggest guidelines regarding acceptable fit criteria: RMSEA ≤ 0.08, SRMR ≤ 0.09, TLI ≥ 0.90, CFI ≥ 0.90. n = 1,928*.

We then proceeded to cross-validate the optimized model (Model 4) in the other English samples, and to verify measurement invariance across the respective groups. As shown in Table 4, fit was excellent for all of the concerned groups, thereby confirming the structure found during our optimization procedure. We do note that while all residual correlations were statistically significant in the Oceania sample, that is not the case in all English samples. While this could be explained, at least in part, by differences in sample size, this suggests that correlating all relevant residuals might not be the most parsimonious approach to achieving fit in all circumstances. Nevertheless, because the inclusion of these residual correlations is theoretically motivated, we contend that they should be maintained. In spite of differences in statistical significance for residual correlations, strict invariance is found across English samples, as shown in Table 4.

**Table 4:** Cross-validation of the optimized model (Model 4) in the other English samples.

**Model**	**Robust X2**	**df**	**Robust CFI**	**Robust TLI**	**Robust RMSEA**	**SRMR**	**BIC**	**AIC**
English sample	581.923	39	0.976	0.959	0.059	0.033	152935.782	152615.016
Oceania	289.078	39	0.977	0.961	0.058	0.031	73600.289	73316.513
North America	209.316	39	0.97	0.949	0.068	0.05	36811.396	36563.396
Europe	135.135	39	0.979	0.965	0.056	0.03	30244.005	30006.447
Asia	94.392	39	0.95	0.915	0.081	0.044	8426.337	8254.198
Configural fit	739.193	156	0.974	0.957	0.062	0.037	149418.274	148140.554
Metric fit	784.362	183	0.974	0.962	0.058	0.039	149234.872	148126.262
Scalar fit	952.825	210	0.967	0.959	0.06	0.041	149186.307	148246.808
Strict fit	996.624	246	0.967	0.965	0.056	0.042	149012.399	148298.379

**Figure 1:**
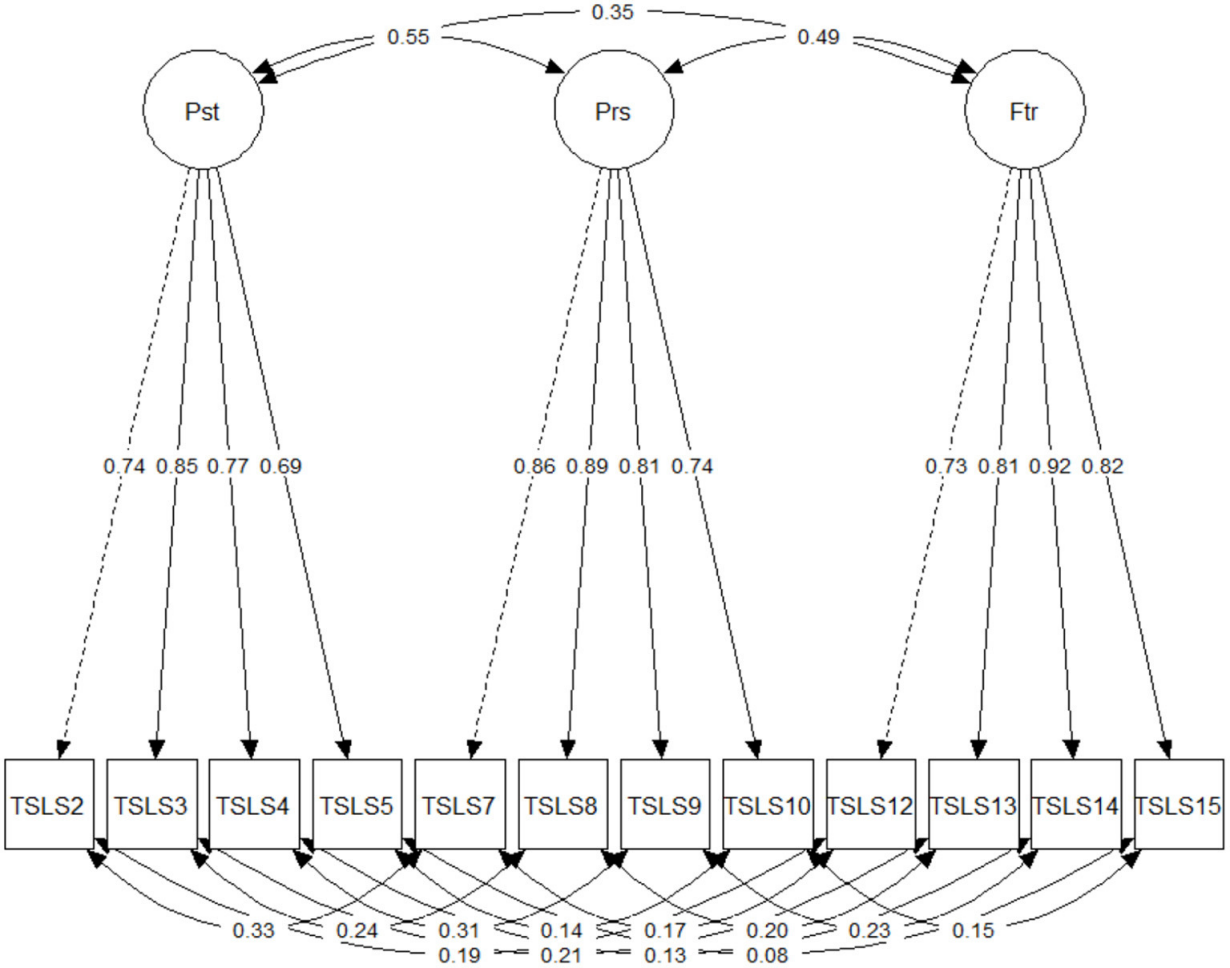
Final 12-item model with full sample. Residuals were suppressed to favor ease of reading.

Finally, we verified measurement invariance across translations. As a preliminary step to this analysis, we tested the measurement model for each translation. As shown in Table 5, acceptable fit is found for all translations except for Finnish, where the TLI is somewhat low (an examination of modification indices for the Finnish sample suggested that fit might benefit from loading item 12 on all three factors). When we turned to the evaluation of measurement invariance, we found that the reduction in CFI at the Metric invariance step was slightly larger than Cheung and Rensvold's (2002) suggested cut-off (the difference in CFI that we report is 0.011). However, because our analysis did include multiple groups, a more liberal cut-off would be justifiable (Rutkowski and Svetina, 2014). In light of this, and of the proximity of our result to Cheung and Rensvold's cut-off, we judge that Metric invariance is an acceptable conclusion. Scalar and Strict invariance are not found across translations. The final measurement model (Model 4), using the data of the full sample, is illustrated in Figure 1.

**Table 5:** Model fit for the measurement model with each language translation.

**Model**	**Robust X2**	**df**	**Robust CFI**	**Robust TLI**	**Robust RMSEA**	**SRMR**	**BIC**	**AIC**
Full sample	1090.071	39	0.974	0.956	0.059	0.037	294231.349	293877.503
English	581.923	39	0.976	0.959	0.059	0.033	152935.782	152615.016
Hungarian	314.306	39	0.958	0.929	0.079	0.051	42846.796	42589.998
Spanish	125.133	39	0.969	0.947	0.056	0.055	26484.409	26252.816
Finnish	153.727	39	0.932	0.885	0.094	0.069	12791.896	12597.375
Slovene	132.349	39	0.945	0.907	0.091	0.087	10607.401	10420.591
Czech	66.05	39	0.980	0.967	0.053	0.031	8928.617	8749.023
Chinese	49.456	39	0.992	0.986	0.034	0.041	8778.385	8603.488
Configural fit	1414.382	273	0.97	0.949	0.065	0.043	264270.548	261828.306
Metric fit	1895.912	327	0.959	0.942	0.07	0.058	264358.569	262285.742
Scalar fit	3140.663	381	0.928	0.912	0.086	0.069	265332.95	263629.538
Strict fit	3860.815	453	0.911	0.909	0.087	0.068	265801.752	264590.892

After obtaining the final model and concluded metric invariance between groups, internal consistency of the 12-item version for the total sample (*n* = 6,662) is as follow: past life satisfaction α = 0.85, ω = 0.85, 95% CI [0.85, 0.86], SE = 0.00, present life satisfaction α = 0.90, ω = 0.90, 95% CI [0.89, 0.90], SE = 0.00, future life satisfaction α = 0.89, ω = 0.89, 95% CI [0.87, 0.90], SE = 0.00.

As an additional verification in the multilingual samples, we explored whether the 12-item model fit the data better than a 15-item model (with the theoretically based residual correlations permitted in both models). Model fit indices for the 15-item model are provided in the supplemental materials. As can be gleaned from the comparison between the table in the supplemental material and Table 4, all fit indices except for one are better in the 12-item model than in the 15-item model (the RMSEA for the Finnish sample is slightly better in the 15-item model). Therefore, while it remains possible that the 12-item model with correlated residuals may not fully optimize fit in all samples, it does improve overall fit over a 15-item model for all languages in the study.

### Correlates With Wellbeing and Illbeing

To further explore the psychometric properties of the TSWLS's structural model found above, Pearson correlations between factor scores of past, present, and future dimensions of the 12-item TSWLS were computed with aspects of wellbeing and illbeing for the full sample. As we obtained metric invariance between our translations, we believe it is appropriate to combine the full sample all together as we now know that factor structure and loadings are equivalent between versions. The following Table 6 shows the results of this analysis.

**Table 6:** Correlations between factor scores of the temporal dimensions of the 12-item TSWLS, and wellbeing and illbeing indicators for the full sample.

			**Temporal satisfaction with life scale**		
**Variable**	***M***	***SD***	**Past**	**Present**	**Future**
**Temporal satisfaction with life scale**				
Past (factor scores)	0.00	1.18	1		
Present (factor scores)	0.00	1.35	0.58	1	
Future (factor scores)	0.00	0.96	0.37	0.52	1
**Strengths use and current knowledge scale**					
Use	26.56	5.38	0.32	0.43	0.40
Knowledge	27.48	4.59	0.28	0.36	0.32
Total	54.04	9.13	0.34	0.44	0.40
**Subjective happiness scale**					
Subjective happiness	4.84	1.27	0.44	0.59	0.44
**Happiness measure**					
Emotional wellbeing **(Fordyce)**	7.63	2.18	0.28	0.41	0.30
**Gratitude questionnaire**					
Gratitude	35.05	5.89	0.32	0.44	0.35
**Adult hope scale**					
Agency	24.42	4.99	0.40	0.54	0.44
Pathway	24.60	4.61	0.23	0.35	0.38
Total	49.03	8.72	0.35	0.50	0.46
**Meaning in life questionnaire**					
Presence	25.10	6.85	0.29	0.44	0.39
Search	22.26	7.81	−0.13	−0.21	−0.03
**Rumination**					
Rumination	25.43	9.08	−0.27	−0.33	−0.20
**Center for epidemiological studies depression scale**					
Depression	14.69	11.12	−0.36	−0.51	−0.32

*n = 6,662*.

*Correlations were done with the factor scores of the optimized 12-item TSWLS, all languages combined. All correlations were significant at the 0.01 level. Dark gray shade = 0.50 or greater. Light gray shade = 0.30 or greater*.

Interpretation of the correlations is based on Cohen's (1988) guidelines where the strength of the correlation is considered small between *r* = 0.10 and *r* = 0.29, medium between *r* = 0.30 and *r* = 0.49 and large when *r* ≥ 0.50. All correlations between the TSWLS and its subscales and wellbeing and illbeing measures were found to be significant at the 0.01 level. As indicated in Table 6, all but one of the possible 39 correlations with wellbeing and illbeing were either small, medium or large; with four being large. Also, the strongest correlations were with present, then future, then past life satisfaction.

## Discussion

The current study aimed to assess the psychometric properties of the Temporal Satisfaction with Life Scale (TSWLS; Pavot et al., 1998), specifically investigating the scale structure and number of optimal items by testing for measurement invariance between subsets of our sample. Furthermore, we aimed to provide correlations between temporal dimensions of life satisfaction and aspects of wellbeing and illbeing on a large multicultural sample.

Firstly, we expected to replicate the same three-factor, 15-item structure as the original study by Pavot et al. (1998). This first hypothesis was partially supported by the data as we did find a three-factor structure, but a better fit with 12 items rather than 15. As with past studies, we found item 11 to be quite problematic (McIntosh's, 2001; Ye, 2007; Tomás et al., 2016; Akyurek et al., 2019; Carrillo et al., 2021). After removing item 11, we proceeded to remove items 1 and 6 as well, as they all derive from the same item from the SWLS (Diener et al., 1985). Following the same logic, we also correlated residuals of similar items. Given that the TSWLS past, present, and future dimensions were developed from the same questions (from the SWLS), we believe it is theoretically acceptable and relevant to correlate residuals within this scale.

Secondly, we expected to find strict invariance of the English version of the TSWLS. This hypothesis was supported by the data as factor loadings, factor structure, intercepts and measurement errors were all held constant across participants from Oceania, North America, Europe, and Asia. This suggests that the 12-item English version of the TSWLS is equivalent and valid across geographical regions of the world and can be used with any English-speaking individuals regardless of their country of residence.

Thirdly, data supported our expectation to find at least configural invariance between the different translations of the TSWLS. The Finnish translation revealed slight issues with item 12's factor loadings which suggests this version might benefit from more psychometric work. However, we were able to find metric invariance between the translations, but not scalar or strict invariance. In more concrete terms, this means that the translations of the questionnaire function in much the same way (similar factor structure and factor loadings), but that we need more research to disentangle possible differential item functioning across cultures from actual group differences in temporal life satisfaction means (and measurement error). This reflects that life satisfaction, and its temporal aspects might have different base levels in different cultures, but scores between translations and cultures are still generally comparable as they are founded in the same structure.

Fourthly, we expected to find positive correlations between the temporal dimensions of the TSWLS and wellbeing measures as well as negative correlations with illbeing measures, with the better fitting 12-item version. Our second hypothesis is confirmed as all measures correlated in the expected way. In addition to this fact, the correlations presented provide further valuable information for researchers and practitioners regarding the strengths of various correlations with various facets of wellbeing and with different temporal perspectives of life satisfaction. For example, the aspect of self-acceptance has relatively strong correlations with all three temporal dimensions of life satisfaction, whereas one may argue that the presence of meaning aspect is important to present and future life satisfaction and not so much the past. Additionally, some aspects, such as autonomy, are not strongly related to any temporal dimensions of life satisfaction, although may be more strongly associated with other faces of wellbeing beyond life satisfaction. In Pavot et al.'s (1998) article outlining the development of the TSWLS they explain various reasons why the measure was created and when it is beneficial to use rather than the SWLS—for example when taking a developmental focus. Again, these correlations with wellbeing and illbeing indicators in relation to different temporal perspectives of life satisfaction can aid such work.

As with all research, the present study was subjected to certain limitations. First the sample was mostly female (81.5% female). Second, within the English-speaking participants, the world regions that had enough participants to perform invariance analyses were not so different culturally. More specifically, participants from Oceania, North America and Europe all share a mostly western culture. Invariance of the English version might benefit from invariance analyses between cultures that are more different from one another (i.e., Middle East and North America). Third, from the 15 different TSWLS translations available through the IWS database, only five had enough participants to perform analyses upon. Therefore, the metric invariance found in the current study can only be extended to the Chinese, Finnish, Hungarian, Slovene, and Spanish versions.

Following the results of this study, we suggest future research use a shortened 12-item version of the TSWLS with items 1, 6, and 11 removed. Insofar as language is associated with culture and that cultural differences might explain group differences on temporal life satisfaction, we find that the use of a 12-item, three-factor structure with correlated residuals between related items is a generally appropriate measurement model for the TSWLS.

## Data Availability Statement

The data that support the findings of this study are not openly available due to ethical constraints, however may be made available from the corresponding author upon reasonable request.

## Ethics Statement

The studies involving human participants were reviewed and approved by the Open Polytechnic of New Zealand Ethics Committee. The participants provided their written informed consent to participate in this study.

## Author Contributions

JG: project administration, conceptualization, methodology, investigation, formal analysis, writing—original draft, and writing—review and editing. AJ: data curation, conceptualization, methodology, investigation, formal analysis, writing—original draft, and writing—review and editing. RJ and DL: formal analysis, writing—original draft, and writing—review and editing. All authors contributed to the article and approved the submitted version.

## Conflict of Interest

The authors declare that the research was conducted in the absence of any commercial or financial relationships that could be construed as a potential conflict of interest.

## Publisher's Note

All claims expressed in this article are solely those of the authors and do not necessarily represent those of their affiliated organizations, or those of the publisher, the editors and the reviewers. Any product that may be evaluated in this article, or claim that may be made by its manufacturer, is not guaranteed or endorsed by the publisher.
