# Supplementary material for: An Evaluation of the Psychometric Properties of the Temporal Satisfaction With Life Scale
Source: Front Psychol. 2022 Apr 14;13:795478. doi: 10.3389/fpsyg.2022.795478 (PMC9047356; doi:10.3389/fpsyg.2022.795478)
Supplement: Supplementary file 1 [file Data_Sheet_1.PDF]

Supplementary material

Model fit indices for the 15-item model

Model	Robust X2	df	Robust CFI	Robust TLI	Robust RMSEA	SRMR	BIC	AIC
Full sample	3245.868	72	0.941	0.913	0.076	0.079	374365.167	373928.064
English	1598.828	72	0.948	0.924	0.073	0.065	194725.83	194329.589
Hungarian	701.669	72	0.928	0.895	0.088	0.109	54132.93	53815.708
Spanish	445.169	72	0.902	0.858	0.086	0.093	34324.643	34038.558
Finnish	274.225	72	0.913	0.874	0.092	0.071	16354.865	16114.575
Slovene	247.428	72	0.92	0.883	0.092	0.096	13667.326	13436.56
Czech	205.657	72	0.927	0.894	0.086	0.097	11354.417	11132.565
Chinese	149.074	72	0.95	0.928	0.069	0.106	11228.092	11012.043
