# Supplementary material for: An Evaluation of the Psychometric Properties of the Temporal Satisfaction With Life Scale
Source: Front Psychol. 2022 Apr 14;13:795478. doi: 10.3389/fpsyg.2022.795478 (PMC9047356; doi:10.3389/fpsyg.2022.795478)
Supplement: Supplementary file 2 [file Data_Sheet_2.PDF]

	Hungarian				Spanish				Finnish				Slovene				Chinese			
	α	ω	95% CI	*se*	α	ω	95% CI	*se*	α	ω	95% CI	*se*	α	ω	95% CI	*se*	α	ω	95% CI	*se*
**Temporal satisfaction with life scale (15-item)**																				
Past	.86	.86	[.84, .87]	.01	.80	.80	[.77, .83]	.01	.85	.85	[.82, .87]	.01	.81	.82	[.78, .85]	.02	.84	.85	[.82, .88]	.02
Present	.90	.90	[.89, .91]	.00	.89	.89	[.88, .91]	.01	.90	.90	[.88, .92]	.01	.89	.89	[.87, .91]	.01	.88	.89	[.86, .91]	.01
Future	.78	.81	[.80, .83]	.01	.72	.72	[.68, .76]	.02	.83	.82	[.78, .86]	.02	.84	.85	[.82, .87]	.01	.74	.79	[.73, .84]	.03
Total	.91	.91	[.90, .92]	.00	.88	.87	[.85, .89]	.01	.94	.94	[.93, .95]	.01	.90	.90	[.88, .92]	.01	.90	.90	[.88, .92]	.01
**Temporal satisfaction with life scale (12-item)**																				
Past	.85	.84	[.83, .86]	.01	.80	.81	[.78, .83]	.01	.85	.85	[.81, .88]	.02	.80	.82	[.78, .85]	.02	.88	.88	[.85, .91]	.02
Present	.89	.89	[.88, .90]	.01	.89	.89	[.87, .91]	.01	.89	.89	[.87, .92]	.01	.88	.89	[.86, .91]	.01	.90	.90	[.87, .92]	.01
Future	.86	.87	[.86, .88]	.01	.81	.82	[.79, .85]	.02	.84	.84	[.80, .87]	.02	.90	.90	[.88, .92]	.01	.87	.87	[.82, .90]	.02
**Strengths use and current knowledge scale**																				
Use	.90	.90	[.89, .91]	.01	.87	.88	[.86, .90]	.01	.89	.89	[.87, .91]	.01	.84	.84	[.80, .87]	.02	.85	.85	[.81, .89]	.02
Knowledge	.84	.85	[.83, .87]	.01	.71	.73	[.68, .76]	.02	.84	.85	[.82, .88]	.02	.77	.78	[.72, .84]	.03	.75	.77	[.72, .82]	.03
Total	.91	.92	[.91, .92]	.00	.87	.87	[.86, .89]	.01	.92	.92	[.90, .93]	.01	.86	.86	[.83, .89]	.02	.87	.87	[.84, .90]	.02
**Subjective happiness scale**																				
Subjective happiness	.81	.81	[.79, .83]	.01	.74	.74	[.70, .78]	.02	.49	.66	[.60, .72]	.03	.79	.80	[.76, .84]	.02	.76	.77	[.69, .82]	.03
**Gratitude questionnaire**																				
Gratitude	.77	.77	[.74, .79]	.01	.75	.72	[.67, .78]	.03	.71	.72	[.67, .76]	.02	.71	.72	[.66, .76]	.03	.79	.80	[.74, .84]	.02
**Adult hope scale**																				
Agency	.82	.81	[.79, .83]	.01	.83	.83	[.80, .86]	.01	.83	.83	[.79, .87]	.02	.72	.73	[.64, .80]	.04	.84	.84	[.80, .88]	.02
Pathway	.82	.83	[.80, .84]	.01	.78	.79	[.75, .82]	.02	.68	.68	[.58, .75]	.04	.68	.71	[.64, .77]	.03	.69	.70	[.61, .76]	.04
Total	.88	.88	[.87, .89]	.01	.87	.87	[.85, .89]	.01	.86	.86	[.82, .89]	.02	.78	.78	[.72, .83]	.03	.85	.86	[.83, .89]	.02
**Meaning in life questionnaire**																				
Presence	.92	.92	[.91, .92]	.01	.88	.88	[.86, .90]	.01	.79	.81	[.77, .84]	.02	.89	.89	[.86, .91]	.01	.82	.83	[.78, .86]	.02
Search	.85	.85	[.83, .87]	.01	.90	.90	[.88, .91]	.01	.87	.88	[.85, .90]	.01	.89	.89	[.86, .91]	.01	.86	.86	[.82, .90]	.02

**Rumination**																				
Rumination	.88	.88	[.87, .89]	.01	.84	.85	[.82, .87]	.01	.87	.87	[.84, .90]	.01	.82	.82	[.78, .85]	.02	.86	.86	[.83, .89]	.02
**Centre for Epidemiological Studies Depression scale**																				
Depression	.92	.92	[.91, .93]	.00	.91	.91	[.90, .92]	.01	.92	.93	[.91, .94]	.01	.91	.91	[.89, .93]	.01	.90	.90	[.88, .92]	.01

	**Czech**			
	α	ω	95% CI	*se*
**Temporal satisfaction with life scale (15-item)**				
Past	.80	.80	[.76, .84]	.02
Present	.88	.88	[.85, .90]	.01
Future	.80	.84	[.80, .88]	.02
Total	.90	.90	[.88, .92]	.01
**Temporal satisfaction with life scale (12-item)**				
Past	.77	.78	[.73, .83]	.02
Present	.86	.86	[.82, .89]	.02
Future	.90	.90	[.87, .92]	.01
**Strengths use and current knowledge scale**				
Use	.88	.88	[.84, .90]	.01
Knowledge	.83	.84	[.79, .87]	.02
Total	.90	.90	[.88, .92]	.01
**Subjective happiness scale**				
Subjective happiness	.82	.82	[.76, .86]	.02
**Gratitude questionnaire**				
Gratitude	.79	.80	[.75, .84]	.02
**Adult hope scale**				
Agency	.79	.80	[.75, .84]	.02

Pathway	.78	.79	[.72, .83]	.03
Total	.84	.84	[.80, .88]	.02
**Meaning in life questionnaire**				
Presence	.89	.90	[.87, .92]	.01
Search	.83	.84	[.80, .88]	.02
**Rumination**				
Rumination	.83	.83	[.79, .87]	.02
**Centre for Epidemiological Studies Depression scale**				
Depression	.90	.91	[.88, .93]	.01

Note. Hungarian *n* = 1,136; Spanish *n* = 693; Finnish *n* = 335, Slovene *n* = 288; Chinese n = 228; Czech *n* = 250. The 95% confidence interval and standard error reported refers to the omega coefficient.

	**Oceania**				**North America**				**Europe**				**Asia**			
	α	ω	95% CI	se	α	ω	95% CI	se	α	ω	95% CI	se	α	ω	95% CI	se
**Temporal satisfaction with life scale (15-item)**																
Past	.87	.87	[.86, .88]	.01	.85	.85	[.83, .87]	.01	.85	.85	[.83, .87]	.01	.80	.80	[.75, .84]	.02
Present	.91	.91	[.91, .92]	.00	.91	.91	[.90, .92]	.01	.90	.90	[.89, .91]	.01	.89	.89	[.86, .92]	.01
Future	.86	.87	[.86, .88]	.01	.85	.86	[.85, .88]	.01	.85	.86	[.85, .88]	.01	.81	.82	[.77, .86]	.02
Total	.92	.91	[.91, .92]	.00	.91	.91	[.90, .92]	.00	.91	.91	[.89, .92]	.01	.87	.87	[.84, .90]	.01
**Temporal satisfaction with life scale (12-item)**																
Past	.86	.86	[.85, .87]	.01	.85	.86	[.84, .87]	.01	.86	.86	[.84, .87]	.01	.80	.81	[.76, .85]	.02
Present	.90	.91	[.90, .91]	.00	.90	.90	[.89, .91]	.01	.89	.89	[.88, .91]	.01	.89	.89	[.86, .91]	.01
Future	.91	.91	[.90, .92]	.00	.91	.91	[.90, .92]	.01	.90	.90	[.89, .82]	.01	.90	.90	[.87, .92]	.01
**Strengths use and current knowledge scale**																

Use	.89	.89	[.88, .90]	.01	.88	.88	[.87, .90]	.01	.88	.89	[.87, .90]	.01	.86	.86	[.82, .90]	.02
Knowledge	.78	.79	[.77, .81]	.01	.76	.76	[.73, .80]	.02	.75	.75	[.71, .78]	.02	.70	.70	[.61, .77]	.04
Total	.89	.90	[.89, .90]	.00	.89	.89	[.88, .91]	.01	.88	.88	[.86, .89]	.01	.87	.87	[.83, .90]	.02
**Subjective happiness scale**																
Subjective happiness	.86	.86	[.85, .88]	.01	.86	.86	[.84, .88]	.01	.82	.82	[.80, .85]	.01	.75	.77	[.71, .82]	.03
**Gratitude questionnaire**																
Gratitude	.84	.83	[.82, .85]	.01	.84	.84	[.81, .86]	.01	.80	.80	[.77, .82]	.01	.77	.76	[.69, .80]	.03
**Adult hope scale**																
Agency	.83	.83	[.82, .85]	.01	.84	.84	[.82, .86]	.01	.81	.81	[.78, .84]	.01	.79	.80	[.72, .85]	.03
Pathway	.81	.81	[.79, .83]	.01	.83	.83	[.80, .85]	.01	.78	.78	[.75, .81]	.02	.73	.73	[.65, .78]	.03
Total	.88	.88	[.87, .89]	.01	.88	.88	[.87, .90]	.01	.86	.86	[.84, .88]	.01	.83	.83	[.79, .87]	.02
**Meaning in life questionnaire**																
Presence	.91	.91	[.91, .92]	.00	.93	.93	[.92, .94]	.00	.90	.90	[.88, .91]	.01	.87	.87	[.83, .90]	.02
Search	.91	.91	[.91, .92]	.00	.91	.91	[.90, .92]	.01	.91	.91	[.90, .92]	.01	.91	.91	[.88, .94]	.02
**Rumination**																
Rumination	.88	.88	[.87, .89]	.00	.89	.89	[.87, .90]	.01	.87	.88	[.86, .89]	.01	.86	.86	[.83, .89]	.02
**Centre for Epidemiological Studies Depression scale**																
Depression	.92	.93	[.92, .93]	.00	.92	.92	[.91, .93]	.00	.93	.93	[.92, .94]	.00	.90	.90	[.88, .92]	.01

Note. Oceania *n* = 1,928; North America *n* = 956, Europe *n* = 779; Asia *n* = 216. The 95% confidence interval and standard error reported refers to the omega coefficient.
